# Suppurating Appendicitis Opening into the Bladder

**Published:** 1904-02

**Authors:** Enrique Fortun

**Affiliations:** Surgeon of Hospital No. 1, Havana


### Suppurating Appendicitis Opening Into the Bladder.

By DR. ENRIQUE FORTUN,
Surgeon of Hospital No. 1, Havana.

[From *Revista Medica Cubana*, July, 1903.]

Juan G., a Spanish merchant, 37 years old, with evident syphilitic antecedents, began to suffer about two months ago from acute pains in the right iliac pit, while a tumefaction was observed in that region. He became an inmate of a clinic of this city, where his case was diagnosticated as malignant neoplasm. After remaining about 20 days, the patient decided to leave for Spain, and went to a hotel here. He was there taken with violent fever and ague, a temperature 41 degrees C., and the first micturition following this attack showed the presence of a great quantity of pus.

Dr. Parra, who was attending the patient, asked me to assist him. The first symptom to which my attention was called was the dimension and hardness of the liver, with swellings, the massiveness of which continued uninterrupted in connection with that in the iliac pit, in which latter region, an accentuated muscular resistance was observed, though presenting a depressed area at the bottom of which the rim of the hepatic gland could be felt.

Temperature was 38 degrees, pulse between 80 and 90, and the general condition of the patient was rather satisfactory.

The diagnosis offered no doubt in our opinion: suppurating appendicitis with evacuation into the bladder; the urine was extremely fetid and mingled, and it contained a large quantity of pus; coexisting syphilitic cirrhosis of the liver was also a part of the diagnosis. On the following day an incision of about 7 cm. long was made into the middle of the depression observed in the iliac pit. We rapidly reached a perfectly defined cavity, which contained a little pus mixed with mucosities. We washed out the cavity with hydrozone and packed it with iodoform gauze. I dressed the wound the following day. We did not find any connection with the bladder, but we did find the appendix and fecal matter.

A complete cure was accomplished in a month, and during that time the liver decreased considerably in volume. Since the third day after the operation antisyphilitic treatment has been followed. The communication between the abscess cavity and the bladder healed after 12 days of treatment.

